# Novel Antibacterial Peptides Isolated from the Maillard Reaction Products of Half-Fin Anchovy (*Setipinna taty*) Hydrolysates/Glucose and Their Mode of Action in *Escherichia Coli*

**DOI:** 10.3390/md17010047

**Published:** 2019-01-10

**Authors:** Jiaxing Wang, Rongbian Wei, Ru Song

**Affiliations:** 1 Key Laboratory of Health Risk Factors for Seafood of Zhejiang Province, School of Food Science and Pharmacy, Zhejiang Ocean University, Zhoushan 316000, China; 18868005756@163.com; 2 School of Marine Science and Technology, Zhejiang Ocean University, Zhoushan 316000, China; rbwei@zjou.edu.cn

**Keywords:** half-fin anchovy hydrolysates, Maillard reaction products, antibacterial peptide, identification, self-production of hydrogen peroxide, membrane damage

## Abstract

The Maillard reaction products (MRPs) of half-fin anchovy hydrolysates and glucose, named as HAHp(9.0)-G MRPs, were fractionated by size exclusion chromatography into three major fractions (F1–F3). F2, which demonstrated the strongest antibacterial activity against *Escherichia coli* (*E. coli*) and showed self-production of hydrogen peroxide (H_2_O_2_), was extracted by solid phase extraction. The hydrophobic extract of F2 was further isolated by reverse phase-high performance liquid chromatography into sub-fractions HE-F2-1 and HE-F2-2. Nine peptides were identified from HE-F2-1, and two peptides from HE-F2-2 using liquid chromatography-electrospray ionization/multi-stage mass spectrometry. Three peptides, FEDQLR (HGM-Hp1), ALERTF (HGM-Hp2), and RHPEYAVSVLLR (HGM-Hp3), with net charges of −1, 0, and +1, respectively, were synthesized. The minimal inhibitory concentration of these synthetic peptides was 2 mg/mL against *E. coli*. Once incubated with logarithmic growth phase of *E. coli*, HGM-Hp1 and HGM-Hp2 induced significant increases of both extracellular and intracellular H_2_O_2_ formation. However, HGM-Hp3 only dramatically enhanced intracellular H_2_O_2_ production in *E. coli*. The increased potassium ions in *E. coli* suspension after addition of HGM-Hp1 or HGM-Hp2 indicated the destruction of cell integrity via irreversible membrane damage. It is the first report of hydrolysates MRPs-derived peptides that might perform the antibacterial activity via inducing intracellular H_2_O_2_ production.

## 1. Introduction

Antibiotics play crucial roles in saving lives and improving human and animal health. However, bacterial pathogens commonly develop antimicrobial resistance due to the extensive use of the antibiotics [1]. As an effective first line of defense against invading pathogens, antimicrobial peptides (AMPs) play a crucial role on the innate immune systems of organisms. The significant advantage of AMPs is their strong antibacterial activity against a very broad spectrum of microorganisms and low rates of bacterial resistance [2]. Natural AMPs can be isolated and characterized from practically all-living organisms [3]. Marine organisms are a good source of AMPs. Recently, an increasing number of AMPs have been isolated from various protein hydrolysates of marine organism sources. For instance, a peptide, CgPep33, rich in cysteine residue was isolated from oyster muscle hydrolysates [4]. Series of short peptides were derived from *Barbel* muscle protein hydrolysates and *Sardinella* (*Sardinella aurita*) hydrolysates [5,6]. One decapeptide GLSRLFTALK was separated from protein hydrolysates of anchovy cooking wastewater [7]. The antibacterial activity of AMPs is related with many factors, such as molecular weight, net charges, hydrophobic domains, and specific amino acid residues in sequences [8].

Maillard reaction (MR), also defined as a non-enzymatic browning reaction, usually occurs in thermal food processing or food storage between amino (amino acid, peptide or protein) and carbonyl groups (e.g. reducing sugar) [9]. The antibacterial activities of some Maillard reaction products (MRPs) or MRP-rich foods, such as chitosan-glucosamine MRPs [10], ε-polylysine-chitosan MRPs [11], xylan-chitosan MRPs [12], roast bread [13], and coffee [14], have attracted great attention in recent years. However, the antibacterial mechanism of MRPs is not yet fully understood. Rufian-Henares and Cueva (2009) reported that coffee melanoidins could exhibit antibacterial activity through metal-chelating of membrane [15]. The bacteriostatic or bactericidal effects of coffee melanoidins were observed at low and high concentrations [16]. Recently, Mueller et al. (2011) identified hydrogen peroxide (H_2_O_2_) generated in coffee brew as a major antibacterial component of coffee [17]. In our previous study, we reported the broad spectrum of half-fin anchovy hydrolysates (HAHp) against food spoilage bacteria [18]. In addition, we found a dramatic increase of extracellular or intracellular H_2_O_2_ formation in *Escherichia coli* (*E. coli*) cells after incubation with the HAHp-derived MRPs (HAHp(9.0)-G MRPs) [19].

H_2_O_2_ is an example of a reactive oxygen species [20]. The generated H_2_O_2_ from MRPs can oxidize almost every compound in the cell, resulting in internal damage associated with increase of membrane permeability [21]. However, to our best knowledge, few studies are focused on the antibacterial activity of peptides isolated from protein hydrolysates MRPs. In addition, the contribution of H_2_O_2_ formation induced by MRP-derived peptides to the antibacterial activity is still not fully understood. For this reason, in this study, HAHp(9.0)-G MRPs were isolated to identify peptides with antibacterial activity and the capacity of H_2_O_2_ self-production. Furthermore, the effects of identified peptides on intracellular and extracellular H_2_O_2_ accumulation on logarithmic phase of *E. coli* cells were investigated to reveal the contribution of H_2_O_2_ formation to the antibacterial effect, inducing by addition of antibacterial peptides.

## 2. Results and Discussion

### 2.1. Purification of Antibacterial and H_2_O_2_ Self-Produced Peptidic Fraction

**Figure 1:**
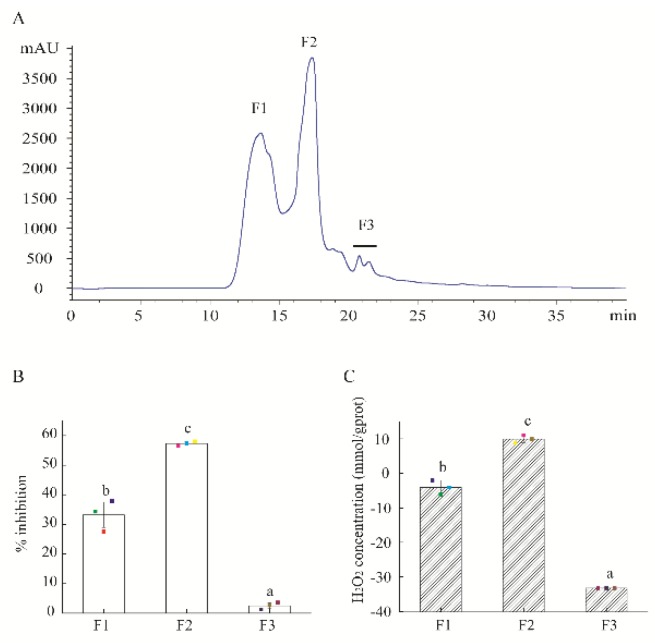
Isolation of HAHp(9.0)-G MRPs and activity of separated fractions. (**A**) The size exclusion chromatography (SEC) of MRPs isolated by high performance liquid chromatography (HPLC) method, detected at 220 nm. (**B**) The percentage inhibition of isolated fractions against *E. coli* cells. (**C**) H_2_O_2_ self-produced concentration of isolated fractions. The actual peptide concentration of isolated fractions was 0.18 mg/mL in the percentage inhibition and H_2_O_2_ production assays. Spots in (**B**,**C**) represent the raw data. The results are expressed as the mean ± standard deviation (*n* = 3). Different letters (a–c) in (**B**,**C**) represent significant differences among isolated fractions (*p* < 0.05).

HAHp(9.0)-G MRPs were separated into three fractions (F1–F3) by HPLC system (Figure 1A). At the actual peptide concentration of 0.18 mg/mL, the antibacterial activity of F2 reached 57.29% against the growth of *E. coli* cells, significantly stronger than the other two fractions (33.22% for F1 and 2.50% for F3) (*p* < 0.05) (Figure 1B). The most bioactive fraction F2 also had the strongest H_2_O_2_ self-production capacity among these fractions (Figure 1C). In recent years, much more attention has been paid to the antibacterial activity of H_2_O_2_ in MRPs. For example, Hauser et al. [21] reported the generated H_2_O_2_ (about 100 μM) in a polyethylene film coated with an active fraction derived from the ribose-lysine MRPs resulted in a log-reduction of >5 log-cycles against *E. coli*. In the present study, the fraction of F2 was collected for further purification.

**Figure 2:**
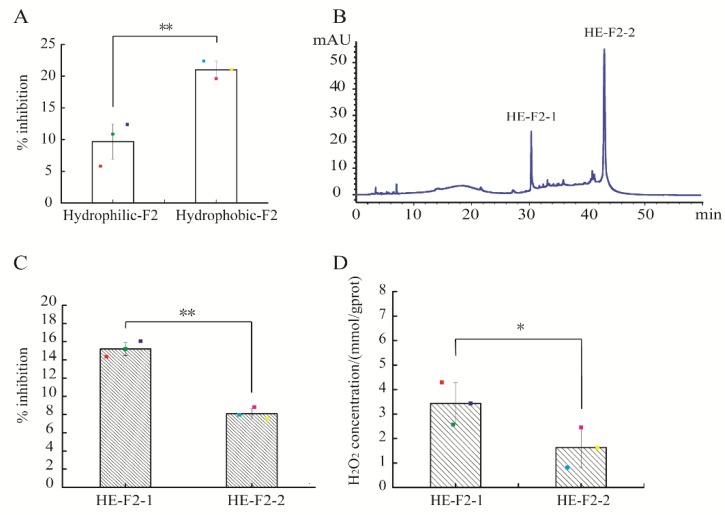
Purification of active fraction F2 using reverse phase high performance liquid chromatography (RP-HPLC) and the activities of sub-fractions assay: (**A**) percentage inhibition of hydrophilic and hydrophobic extracts of F2; (**B**) chromatogram of active fraction F2 by RP-HPLC, measured at 280 nm; (**C**) percentage inhibition of F2-1 and F2-2; and (D) H_2_O_2_ production capacity of F2-1 and F2-2. Spots in (**A**,**C**,**D**) represent the raw data. The results are expressed as the mean ± standard deviation (*n* = 3). The symbol of “**” and “*” in (**A**,**C**,**D**) represent significant differences of *p* < 0.01 and *p* < 0.05, respectively.

Through Cleanert^®^ S C18-N solid phase extraction, the active fraction F2 was further isolated into hydrophilic and hydrophobic extracts. At the actual peptide concentration of 0.15 mg/mL, the percentage inhibition of hydrophobic extract of F2 against *E. coli* was 20.98 ± 1.39%, remarkably higher than that of the hydrophilic counterpart (11.61 ± 2.27%) (*p* < 0.05) (Figure 2A). Therefore, the hydrophobic extract of F2 (HE-F2) was selected for further purification using a C_18_ column (4.6 × 250 mm, 5 μm) based on the hydrophobic property of peptides. As shown in Figure 2B, HE-F2 was separated into two major peaks, HE-F2-1 and HE-F2-2. HE-F2-1 demonstrated stronger antibacterial activity than HE-F2-2 (*p* < 0.01) (Figure 2C). A similar result was found for the self-produced H_2_O_2_ concentrations in HE-F2-1 and HE-F2-2 (*p* < 0.05) (Figure 2D). The results in Figure 1; Figure 2 suggest that the self-production of H_2_O_2_ could be an important contributor for the observed antibacterial activity of active peptidic fractions derived from HAHp(9.0)-G MRPs.

### 2.2. Identification of Peptide by Liquid Chromatography–Electrospray Ionization/Multi-Stage Mass Spectrometry (LC-ESI-Q-TOF-MS/MS)

HE-F2-1 and HE-F2-2 were subjected to LC-ESI-Q-TOF-MS/MS analysis to identify all potential peptides. The molecular mass of peptide was identified according to its proton charged [M + H]H^+^ precursor ion. A few peptides were matched with actin cytoplasmic 1 (*Clupea harengus*) in the protein database of *Clupeoidei* after searching in NCBI; however, the −10lgP scores of these peptides were below 35 (data not shown), suggesting low confidence for these matched peptides. Therefore, in this study, we used de novo analysis to identify potential peptides. After collapses of the precursor ion into series fragments, a single peptide fragment could be auto-matched by de novo peptides sequencing [22]. Peptides with average local confidence scores (ALC) ≥ 95% and local confidence of each residue in peptide sequence ≥ 90% in HE-F2-1 and HE-F2-2 through de novo peptide automated spectrum processing are listed in Table 1.

The mass of identified peptides in HE-F2-1 and HE-F2-2 ranged from 700 Da to 1700 Da. Peptide sequences analysis in the antimicrobial peptide database (APD) database (http://aps.unmc.edu/AP/main.html) and NCBI non-redundant peptide database (http://www.ncbi.nlm.nih.gov/blast) indicated that no AMPs or peptides had the identical sequences with these identified peptides herein. In this study, these identified peptides had different sequences. Nevertheless, nine peptides, namely KGTAVPTAAEATAQR, FEDQLR, SVVMLR, LDVLADK, EGDALDELR, EAGAEFDKAAEEVKR, MEVLLLER, VATVSLPR, and RHPEYAVSVLLR, had common cationic arginine (R) or lysine (K) residues at the N- or C-terminus of sequences. In addition, the other peptides, ALERTF and LLDRLPRPL, had R residues within sequences. The cationic R or K residues in the sequences of identified peptides were consistent with some published studies, such as peptides RKSGDPLGR and AKPGDGAGSGPR derived from protamex hydrolysates of Atlantic mackerel byproducts [23], and a synthetic peptide VRRFPWWWPFLRR with a wide antibacterial spectrum [24]. The presence of positively charged residues at the N- or C-terminus could contribute to the interaction with negatively charged phospholipids present on *E. coli* membrane surface [8]. Similarly, we identified seven peptides (RVAPEEHPTL, WLPVVR, FFTQATDLLSR, VLLLWR, VLLVLLR, VLLALWR, and LLSWYDNEFGYSNR) from HAHp(9.0)-G MRPs that had R residues at the N- or C-terminus [25]. In consideration of the characteristics of peptide sequences in Table 1, it was quite apparent that the presence of R residue, especially at C- or N- terminus, could be a typical property for the peptides derived from HAHp(9.0)-G MRPs.

### 2.3. Physicochemical Property of Synthetic Peptides

**Figure 3:**
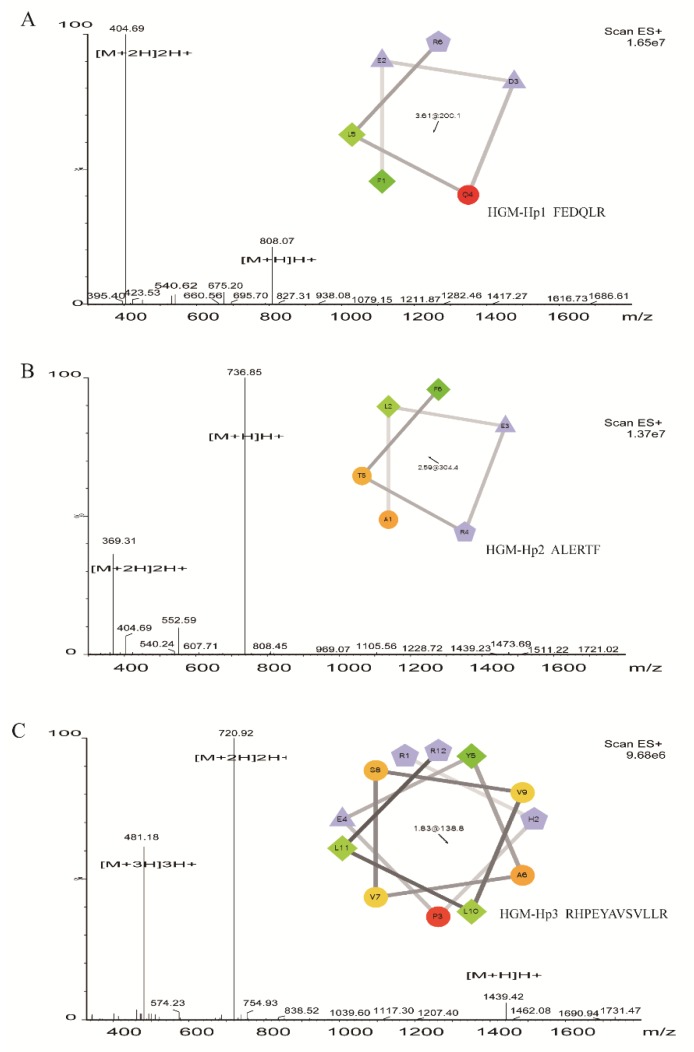
ESI-MS and helical wheel projection of synthetic peptides: (**A**) HGM-Hp1, FEDQLR; (**B**) HGM-Hp2, ALERTF; and (**C**) HGM-Hp3, RHPEYAVSVLLR. The helical wheel projection of HGM-Hp1, HGM-Hp2, and HGM-Hp3 (insert diagrams in Figures (**A**–**C**)) were performed using the online website tool (http://rzlab.ucr.edu/scripts/wheel/wheel.cgi). In helical wheel projection, circles and diamonds represent hydrophilic and hydrophobic residues, respectively. The green color, whose intensity decreases proportionally to the hydrophobicity, represents the most hydrophobic residue. Non-hydrophobic portions are encoded in yellow. The red color is used to encode hydrophilic residues, whose intensity represents the extent of hydrophilicity. The charged residues are encoded in light blue. Negatively charged and positively charged residues are displayed as triangles and pentagons, respectively. The hydrophobic moment is denoted in the center.

Considering the presence of R residue in peptide sequences related with antibacterial activity, F or Y residue in peptide sequence consistent with the specific absorbance of HE-F2-1 or HE-F2-2 at 280 nm, and the intensity of identified peptides, we selected peptide FEDQLR derived from HE-F2-1 (named as HGM-Hp1), and peptide RHPEYAVSVLLR from HE-F2-2 (named as HGM-Hp3) for synthesis. Besides R and F residues in sequence, peptide ALERTF was the only one in Table 1 with net charge of 0 (see Table 2), therefore peptide ALERTF was also synthesized (named as HGM-Hp2). The purity (>98%) of the peptides was verified by RP-HPLC. The properties of ESI-MS and helical wheel projection of the three synthetic peptides are shown in Figure 3.

Based on the precursor ion of [M + H]H^+^, the measured molecular weights of HGM-Hp1, HGM-Hp2 and HGM-Hp3 were calculated as 807.07 Da, 735.85 Da, and 1438.42 Da, respectively, which were in very good agreement with their theoretical values (see Table 1). The ESI-MS results in Figure 3 suggest that the peptides were successfully synthesized. The property of α-helices in protein or peptide can be observed in the plot of wheel projection [26]. Usually, hydrophobic amino acids are concentrated on one side of the helix, and polar or hydrophilic amino acids are located on the other side [27]. According to the wheel projection, HGM-Hp1 possessed hydrophobic F_1_ and L_5_ residues on the hydrophobic side, while the charged residues E_2_D_3_ and R_6_ were observed on the opposite side (diagram in Figure 3A). In the projection diagram of HGM-Hp2, the hydrophobic L_2_ and F_6_ residues were concentrated on the hydrophobic side, and the negatively charged E_3_ and positively charged R_4_ residues were located on the opposite side (diagram in Figure 3B). In contrast, HGM-Hp3 might form α-helices in the wheel projection due to more hydrophobic and hydrophilic residues concentrated on the opposite sides (diagram in Figure 3C).

To further reveal the characteristics of these synthetic peptides, their physicochemical properties were compared, as summarized in Table 2.

HGM-Hp3 had larger molecular weight than the other two peptides. The *pI* value of 4.00 and net charge of −1 for peptide HGM-Hp1 indicated its acidic characteristics. By comparison, the positively charged property was predicted in peptide HGM-Hp3 according to its *pI* value of 9.53 and net charge of +1. GRAVY is associated with the hydrophobicity of peptide or protein, which is calculated by the sum of hydropathy values of all amino acids divided by the peptide or protein length [28]. The negative and positive values of GRAVY suggest corresponding hydrophilic and hydrophobic properties of peptides [25]. In this sense, the negative GRAVY values of HGM-Hp1 (−1.400), HGM-Hp2 (−0.050), and HGM-Hp3 (−0.133) indicated potential hydrophilic property of these peptides. Furthermore, the hydrophobicity, as measured by an experimentally determined Wimley–White scale and associated with the free energy transitioning a peptide from aqueous environment to a hydrophobic environment, was similar for HGM-Hp1 (+14.79 Kcal/mol) and HGM-Hp3 (+14.45 Kcal/mol). The ProtParam Tool predicted the instability indexes of HGM-Hp1 and HGM-Hp3 were 72.53 and 73.08, which belong to the unstable peptide category. The secondary structures of the three synthetic peptides were predicted to form 100% random-coil structures obtained from the NPS^@^SOPMA secondary structure prediction.

Compared with the traditional AMPs with > 50 amino acid residues, all of the synthetic peptides in this study are small (<1.5 kDa) and have a substantial portion of hydrophobic residues. The random coil structures were predicted for the three synthetic peptides (Table 2), although the helical wheel projection of these peptides are observed in Figure 3. Generally, peptides without disulfides are often in disorder when dissolved in aqueous solutions [29,30]. However, the AMPs with a random coil structure can be structured in aqueous solution once contacted with biological membrane or dissolved in hydrophobic environment, which could contribute to the binding with membrane, or occurrence of self-aggregation [31].

**Figure 4:**
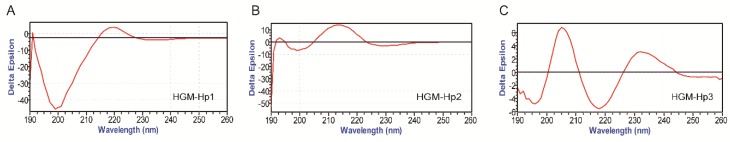
CD spectra of synthetic peptides in membrane-mimicking solution (1.6 mmol/L sodium dodecyl sulfate (SDS), dissolved in 10 mmol/L PBS, pH 7.4): (**A**) HGM-Hp1; (**B**) HGM-Hp2; and (**C**) HGM-Hp3. The peptide concentration was 0.5 mg/mL.

The actual secondary structures of these synthetic peptides under membrane mimic solvents were analyzed using circular dichroism (CD) measurements, as shown in Figure 4.

The negative band at 200 nm, due to random coil structure [32], was observed in HGM-Hp1 and HGM-Hp2. However, besides the negative band at 200 nm, other positive CD signals, such as a weak peak at 191 nm and a broad band at 219 nm, were noticed in the CD spectrum of HGMp1. Similarly, two positive bands at 193 nm and 214 nm were found in the CD spectrum of HGMp2. The results in Figure 4A,B suggest that peptides HGM-Hp1 and HGM-Hp2 could form random coil and other structures upon interaction with membrane-mimicking solvents. By comparison, the peptide HGM-Hp3 may tend to form type I β-turn structure in the membrane-mimicking solvents, with a negative band at 196 nm, and a positive band at 205 nm [33]. Protein or peptide with a maximum at 202 nm and a minimum at 216 nm in the CD spectrum could adopt a β-sheet conformation [34]. As shown in the CD spectrum of HGMp3 (Figure 4C), a positive band at 205 nm and a negative band at 218 nm may indicate some kind of β-sheet structure formation.

### 2.4. Antibacterial Activity and H_2_O_2_ Self-Production of Synthetic Peptides

**Figure 5:**
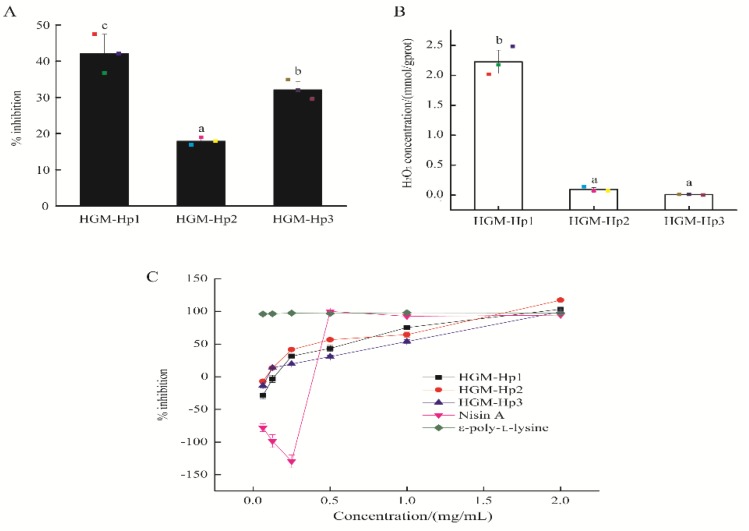
Antibacterial activity and H_2_O_2_ self-production of synthetic peptides: (**A**) percentage inhibition against *E. coli* cells; (**B**) H_2_O_2_ self-production of synthetic peptides at the actual peptide concentration of 0.25 mg/mL; and (**C**) percentage inhibition of synthetic peptides, nisin A and ε-poly-lysine against *E. coli* at different concentrations. Raw data in (**A**,**B**) are displayed as spots. The results are expressed as the mean ± standard deviation (*n* = 3). Different letters (a–c) in (**A**,**B**) indicate significant differences among samples (*p* < 0.05).

The antibacterial activity and self-production of H_2_O_2_ of the three synthetic peptides were compared, as shown in Figure 5.

At the actual peptide concentration of 0.25 mg/mL, HGM-Hp1 demonstrated the highest percentage inhibition (42.10 ± 5.36%), in contrast to HGM-Hp2 (17.93 ± 1.03%) and HGM-Hp3 (32.14 ± 2.19%) (*p* < 0.05) (Figure 5A). In addition, the peptide HGM-Hp1 exhibited the highest H_2_O_2_ self-production capacity among the three synthetic peptides (*p* < 0.05) (Figure 5B). All synthetic peptides showed concentration-dependent inhibition of the growth of *E. coli* cells, as shown in Figure 5C. In addition, no growth of *E. coli* cells was observed after treatment with the synthetic peptides at the actual peptide concentration of 2 mg/mL. Therefore, the MIC was 2 mg/mL for peptides HGM-Hp1, HGM-Hp2, and HGM-Hp3 against the growth of *E. coli*. By comparison, nisin A, an AMP from *Lactococcus lactis*, had negative inhibition effects on *E. coli* cells at the concentration below 0.5 mg/mL. The antibacterial effect of nisin A reached a plateau (100% inhibition) when its concentration was increased to 0.5 mg/mL. Similar to our results, Tong et al. [1] reported the MIC of nisin against *Enterococcus faecalis* was 1 g/L (actual concentration of 0.5 mg/mL). Natural ε-poly-lysine has a broad antimicrobial activity against Gram-negative and Gram-positive bacteria [35]. In this study, the percentage inhibition on *E. coli* was 95.99% after treatment with 62.5 μg/mL of ε-poly-lysine. This result was in agreement with the MIC of ε-poly-lysine solution, which was less than 100 μg/mL against *E. coli* [36]. ε-poly-lysine consists of 25–30 positively charged L-lysine residues, which are responsible for its strong antibacterial activity [35].

As an anionic peptide, HGM-Hp1 (FEDQLR), with the positively charged residue R at the C-terminus, may not contribute to the interaction with the negatively charged bacterial membrane surface. We speculated specific residues in HGM-Hp1 could be associated with its antibacterial activity. By searching the AMPs in APD database, we found HGM-Hp1 (FEDQLR) had the same two residues (ED) in the sequence as some reported anionic peptides, such as peptides DEDLDE (net charge: −5) and DEDDD (net charge: −5) isolated from *Xenopus laevis* skin [37,38], and the surface-tethered peptide GATPEDLNQKLS (net charge: −1) [38]. The more amphiphilic and smaller is the peptide, the more it diffuses through the membrane to exert bacteriostatic effect [39]. The smallest molecule size of HGM-Hp2 might be a significant contributor to its inhibitory effect against *E. coli*.

HGM-Hp3 had a relatively higher molecular weight than HGM-Hp1 or HGM-Hp2 (see Table 2), which might be an inconvenient factor for its quick diffusion in biomembrane. Nonetheless, the sequence property of HGM-Hp3 (RHPEYAVSVLLR), such as the cationic net charge, the presence of positively charged residues R at the C- and RH at the N- terminus, and the hydrophobic regions (AV, VLL) in sequence, were consistent with some cationic antibacterial peptides in APD database. For example, Hilpert et al. (2009) [38] reported a surface-tethered cationic peptide RRAAVVLIVIRR (net charge: +4) with R residues at C- and N- terminus, and hydrophobic regions (AAVVLIVI). Kim et al. (2016) [40] identified a cationic peptide LRHKVYGYCVLGP (net charge: +2) from American cockroach *Periplaneta americana* (*Linnaeus*) with positively charged residues (RHK) close to the N-terminus, and hydrophobic regions (VL) in sequence. Obviously, the sequence characteristics of HGM-Hp3 could be responsible for its inhibitory effect on the growth of *E. coli*.

### 2.5. Antibacterial Activity of Synthetic Peptides on the Logarithmic Growth Phase of E. coli and Intracellular and Extracellular H_2_O_2_ Formation

The logarithmic growth phase of *E. coli* cells suspended in saline were added to the synthetic peptide solution. In that case, peptides could easily contact with the newly formed *E. coli* cell membrane. After incubation at 37 °C for 3 h, the percentage inhibition of synthetic peptides on logarithmic phase of *E. coli* was measured, and the induced production of intracellular and extracellular H_2_O_2_ in *E. coli* cells by addition of synthetic peptides were investigated as well.

**Figure 6:**
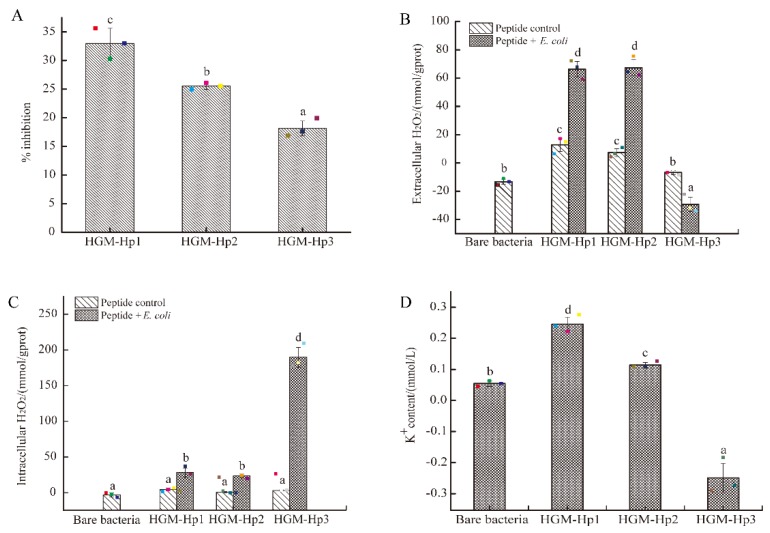
Antibacterial activity of synthetic peptides on the logarithmic growth phase of *E. coli* cells: (**A**) percentage inhibition; (**B**) extracellular H_2_O_2_ concentration; (**C**) intracellular H_2_O_2_ concentration; and (**D**) extracellular potassium ion (K^+^) content. Raw data are displayed as spots. *E. coli* cells and peptides treated under the same conditions were used as the bare bacteria and peptide control, respectively. The results are represented as the mean ± standard deviation (*n* = 3). Different letters (a–d) indicate significant differences among samples (*p* < 0.05).

As shown in Figure 6A, HGM-Hp1 still demonstrated the strongest inhibitory activity on the growth of logarithmic phase *E. coli* cells among the three synthetic peptides. However, the percentage inhibitions of HGM-Hp2 and HGM-Hp3 on logarithmic phase *E. coli* cells were not in accordance with the result of Figure 5A. It was noticed that HGM-Hp2 showed higher percentage inhibition (25.54 ± 0.57%) than HGM-Hp3 (18.15 ± 1.30%) (*p* < 0.05) after incubated with logarithmic phase of *E. coli* cells. Interestingly, after incubation of 3 h at 37 °C, the H_2_O_2_ concentrations of bare HGM-Hp1 and HGM-Hp2 (peptide controls) (Figure 6B) were obviously higher than their H_2_O_2_ self-productions in Figure 5B. Although the incubation of bare peptide at 37 °C contributed to produce more H_2_O_2_, the concentration of extracellular H_2_O_2_ in *E. coli* cells after treatment with HGM-Hp1 or HGM-Hp2 at 37 °C for 3 h was significantly higher than those observed in the bare bacteria or their peptide controls (*p* < 0.05) (Figure 6B).

Likewise, the intracellular H_2_O_2_ concentration in *E. coli* cells after incubation with HGM-Hp1 or HGM-Hp2 were significantly higher than those in the bare bacteria or their peptide controls (*p* < 0.05) (Figure 6C). In contrast, the increase of extracellular H_2_O_2_ production was not observed in the logarithmic phase of *E. coli* cells after treatment of HGM-Hp3 (Figure 6B). Nevertheless, the highest intracellular H_2_O_2_ concentration in *E. coli* cells was induced by HGM-Hp3 addition (189.74 mmol/gprot), which was dramatically increased than those in HGM-Hp1 (28.16 mmol/gprot) or HGM-Hp2 (23.35 mmol/gprot) treatment (*p* < 0.05) (Figure 6C). The results in Figure 6 suggest that peptides HGM-Hp1, HGM-Hp2, and HGM-Hp3 induced intracellular H_2_O_2_ production in logarithmic phase of *E. coli* cells. This result is similar to our previous study of HAHp(9.0)-G MRPs, which induced H_2_O_2_ production in *E. coli* cells after 3 h of incubation at 37 °C [19]. In a recent study, Bucekova et al. (2018) [41] reported the antibacterial activity of honey samples incubated at 45 °C was enhanced by 25% when compared to untreated honeys, and significantly increased H_2_O_2_ accumulation and glucose oxidase activity were detected as well in the honey samples exposed to 45 °C. This means the H_2_O_2_ formation in samples or treated cells could play a crucial role for the antibacterial activity of mild thermal products or MRPs.

However, the H_2_O_2_ production in *E. coli* cells induced by HGM-Hp1 and HGM-Hp2 addition were different from the case of HGM-Hp3. Apparently, the treatments of HGM-Hp1 and HGM-Hp2 quickly resulted in extracellular H_2_O_2_ accumulation in the logarithmic phase of *E. coli* cells, while HGM-Hp3 caused dramatic increases of intracellular H_2_O_2_ accumulation. The aqueous solution of logarithmic phase *E. coli* cells could provide enough cell membranes for interaction with peptides, which could contribute to the rapid permeation of smaller peptides HGM-Hp1 (807.07 Da) and HGM-Hp2 (735.85 Da) into the biomembrane as compared with the relatively large peptide of HGM-Hp3 (1438.42 Da). Furthermore, the dramatic increases of intracellular H_2_O_2_ accumulation in logarithmic phase of *E. coli* cells after addition of synthetic peptides also suggested the undergone oxidative stress induced by these synthetic peptides.

High ROS level in cells can lead to oxidative damage to cell membranes, and finally result in morphological and physiological changes [42,43]. In this context, measuring the efflux of K^+^ from bacterial cells, a classical method to assess the membrane damage caused by antimicrobial agents [44,45,46], was a priority to determine after addition of synthetic peptides. As shown in Figure 6D, the amount of extracellular K^+^ ions in HGM-Hp1 or HGM-Hp2 treatment was significantly higher than that in the bare bacteria (without synthetic peptide addition) (*p* < 0.05). The trend of K^+^ content variations of peptide treatments was consistent with the change of their percentage inhibitions (Figure 6A). The higher the K^+^ content was in the cell suspension after treatment, the more serious was the observed membrane damage. In addition, the results in Figure 6D suggest that the destruction of membrane integrity could contribute to dramatic increase of extracellular H_2_O_2_ in HGM-Hp1 and HGM-Hp2 treatments, due to accumulated intracellular H_2_O_2_ leakage. Based on the result of Figure 6, we supposed that the intracellular H_2_O_2_ accumulation might play an important role on the antibacterial action of synthetic peptides via ROS trigger pathway, especially for shorter peptides such as HGM-Hp1 and HGM-Hp2. To the best of our knowledge, it is the first report of antibacterial peptide derived from the MRPs of marine protein hydrolysates could perform the antibacterial activity via inducing intracellular H_2_O_2_ production.

## 3. Materials and Methods

### 3.1. Materials

Half-fin anchovy (*Setipinna taty*), were bought from the local aquatic market in Zhoushan City, China. The strain of *E. coli* CGMCC 1.1100 used as the indicative bacterium was provided by School of Food Science and Pharmacy, Zhejiang Ocean University. H_2_O_2_ and K^+^ assay kits were purchased from Jiancheng Bioengineering Institute (Nanjing, China). Reagents used in HPLC or RP-HPLC, and MS were chromatographic and mass spectrometric grade, respectively. Other reagents were analytical grade and were commercially available.

### 3.2. Preparation of HAHp(9.0)-G MRPs

HAHp was prepared according to our previous method [18]. In brief, the minced half-fin anchovy was blended with deionized water at a ratio of 1:4 (*w*/*v*). The pH of the mixture was adjusted to 2.0 using 6 mol/L HCl, then preincubated at 37 °C for 30 min. Pepsin was added at a ratio of 1100 U/g and incubated at 37 °C for 2.4 h. The hydrolysis was terminated by heating at 95 °C for 10 min to inactive pepsin. Then, the hydrolysates were adjusted to pH of 5.0 using 6 mol/L NaOH, and centrifuged at 10,000× *g* for 20 min at 4 °C (Himae CF 16 RX versatile compact centrifuge, Tokyo, Japan) to remove insoluble material (including upper layer of fat and sediments). The soluble hydrolysates, namely HAHp, were filtered and adjusted to pH of 9.0. After centrifugation of 10 min at 6000× *g*, the supernatant was blended with glucose at a ratio of 100:3 (v/m), followed by thermal treatment at 120 °C for 100 min. The generated dark brown products, designated as HAHp(9.0)-G MRPs, were kept at −20 °C until use. The mass of peptide in HAHp(9.0)-G MRPs was 6.71 mg/mL. The production rate of peptide in HAHp(9.0)-G MRPs per 100 g content of half-fin anchovy (wet weight) was 2.68%.

### 3.3. Peptide Concentration Determination

Peptide concentration was determined using the method of O-phthalaldehyde (OPA) described by Bougherra et al. (2017) [47] with slight modifications. Briefly, 25 μL of sample were mixed with 1 mL of OPA solution. After completely blending, 600 μL of the mixture solution were added into a micro cuvette. The absorbance was determined at 340 nm with a UV-vis 1200 spectrophotometer (Hitachi, Tokyo, Japan). Glutathione was used as a standard peptide to make standard curve.

### 3.4. Antibacterial Assay

The antibacterial activity of isolated fractions was determined using the method of 96-well micro-plate [18]. Briefly, 100 μL of *E. coli* cells at the logarithmic phase were pipetted into 10 mL of sterilized nutrient broth to prepare the *E. coli* suspension. Then, 50 μL of samples were added with 50 μL of *E. coli* suspension and incubated at 37 °C for 24 h. The absorbance of sample (A_S_) was measured at 630 nm by a SM-800 micro-plate reader (Shanghai Utrao Medical Instrument Co., Ltd, China). The antibacterial activity, indicated as the percentage inhibition, was calculated according to the following equation:

Percentage inhibition (%) = [A_C_ − (A_S_ − A_SB_)]/(A_C_ − A_B_)] × 100 (1)

where A_C_ is the absorbance of control, with the same volume of distilled water instead of sample solution; A_S_ is the absorbance of the sample; A_SB_ is the absorbance of sample blank, with same volume of distilled water in substitute of *E. coli* suspensions; and A_B_ is the absorbance of distilled water.

### 3.5. H_2_O_2_ Assay

The amount of H_2_O_2_ self-production in isolated fractions or peptides was determined using the H_2_O_2_ assay kit. Briefly, 250 μL of Reagent 1 (incubated at 37 °C) were added with 25 μL of sample, and 250 μL of Reagent 2. After complete blending, the absorbance of sample tube (A_S_) was measured at 405 nm using a micro-cuvette. The amount of H_2_O_2_ was calculated according to the following equation:

H_2_O_2_ content (mmol/gprot) = [(A_S_ − A_B_)/(Ac − A_B_)] × 163/P_C_ (2)

where A_B_ is the absorbance of blank tube, using the same volume of double-distilled water instead of the sample; Ac is the absorbance of standard tube, using the same volume of 163 mmol/L H_2_O_2_ instead of the sample; 163 is the concentration of standard H_2_O_2_ solution (mmol/L); and P_C_ is the protein concentration (mg/mL).

### 3.6. Purification of H_2_O_2_ Self-Production Peptides from HAHp(9.0)-G MRPs

#### 3.6.1. SEC

After being filtered through a 0.22 μm micro-filter, the filtration of HAHp(9.0)-G MRPs was used for purification. The isolation of peptidic components was carried out by employing a 1260 Agilent HPLC system equipped with a PL aquagel-OH 30 column (75 × 300 mm, 8 μm, Agilent Technologies, Inc., Santa Clara, CA, USA), with separation mass ranging from 100 to 30,000 Dalton. The injection volume of HAHp(9.0)-G MRPs was 20 μL. The isolation process was firstly eluted with 30% acetonitrile at a flow rate of 0.5 mL/min under temperature of 25 °C and the UV absorption was measured at 220 nm. The purification was repeated 20 times at the same elution conditions to collect enough isolated fractions for further assay. The freeze-dried isolated fractions were dissolved in distilled water. The antibacterial activity (measured as percentage inhibition) and H_2_O_2_ self-production capacity (represented as mmol/gprot) of isolated fractions were assayed. The active peptic fraction was then selected for further purification.

#### 3.6.2. Solid Phase Extraction (SPE) and Purification by RP-HPLC

The active fraction F2 isolated from SEC was separated using a small SPE cartridge (Cleanert^®^ S C18-N, 500 mg/3mL, Agela Technologies, Tianjin, China). Prior to elution with 3 mL of 30% methanol (containing 0.1% formic acid), the SPE cartridge was pre-activated with 6 mL methanol and then rinsed with 12 mL water. The filtrate or eluent were collected and designated as the hydrophilic extract. The absorbed hydrophobic compounds were washed off with 3 mL of 70% acetonitrile. The hydrophilic and hydrophobic components of the extract were lyophilized separately. After being re-dissolved in distilled water, the percentage inhibitions of hydrophilic and hydrophobic components of F2 on the growth of *E. coli* cells were measured by utilizing the 96-well micro-plate method (described in the Section 2.3).

The hydrophobic extract of F2, which showed the largest percentage inhibition, was loaded onto a RP-HPLC system equipped with a C_18_ column (4.6 × 250 mm, 5 μm, Sunfire™, Waters, MA, USA) for further separation. Solvent A, 0.1% (*v*/*v*) trifluoroacetic acid (TFA) in water, and solvent B, 100% acetonitrile with 0.1% (*v*/*v*) TFA, were used for elution. A gradient elution was carried out as follows: 0–25 min, 5–45% solvent B; 25–30 min, 45–85% solvent B; 30–40 min, 85–95% solvent B; 40–50 min, 95–5% solvent B; 50–60 min, 95–5% solvent B, at a constant flow rate of 1.0 mL/min, and measured at 280 nm. Major separated peaks of F2 were collected, then freeze-dried. The percentage inhibitions of major peaks in F2 against *E. coli* cells, as well as the H_2_O_2_ self-production capacity were measured and compared.

#### 3.6.3. Identification of Peptide by LC-ESI-Q-TOF-MS/MS

The bioactive peak derived from RP-HPLC was subjected to LC-ESI-Q-TOF-MS/MS analysis, aiming to identify all potential peptides. The separation was performed on a Nano Aquity UPLC system (Waters Corporation, Milford, MA, USA), coupled with a quadrupole-Orbitrap mass spectrometer (Q-Exactive) (Thermo Fisher Scientific, Bremen, Germany) and equipped with an online nano-electrospray ion source. Elution was carried out by applying the UPLC system, according to the following procedures: 4 μL of sample were loaded on a C_18_ column (75 μm × 250 mm, 3 μm, Eksigent, Livermore, CA, USA). Solvent A (0.1% formic acid in water) and solvent B (acetonitrile with 0.1% formic acid) were used for the gradient elution, 0–45 min, 5–30% solvent B; 45–50 min, 30–80% solvent B; 50–52 min, 80% solvent B; 53 min, 5% solvent B; 53–60 min, 5% solvent B, at a flow rate of 300 nL/min. First order MS detection was performed in a positive MS-mode with resolution of 70,000, and the automatic gain control (AGC) was set at 1 × 10^6^, mass-to-charge ratio scanning from 350 to 1600 m/z. At each elution time, ten strongest ions were automatically selected for MS/MS analysis using a higher energy collisional dissociation (HCD). The resolution of MS/MS was 17,500, and AGC was set at 2 × 10^5^. Raw data files of peptides acquired on the Q-TOF from the samples were searched against the protein database of *Clupeoidei* in NCBI. Through homology analysis in *Clupeoidei*, peptide was considered a match if its −10lgP score was greater than 80. Furthermore, de novo peptide automated spectrum processing was performed using the PEAKS Studio software (7.0) (Bioinformatics Solutions Inc., Waterloo, Canada) (http://www.bioinfor.com). PEAKS Studio awards confidence scores for the entire range of amino acid sequences studied [48]. In de novo peptide, the ALC and local confidence scores indicate that the probability of peptide sequence is correct [48]. In this study, de novo sequences of peptides with ALC ≥ 95%, and local confidence of each amino acid residue ≥ 90% were set for validation of predicted peptides.

### 3.7. Peptide Synthesis

Three peptides were synthesized by Synpeptide Co., Ltd. (Shanghai, China) based on their sequences. The purity of synthetic peptide was determined (>98%) using HPLC analysis. Furthermore, the molecular mass of synthetic peptides was also measured using MS analysis under ESI positive mode. The helical wheel projection of peptide was performed using an online tool (http://rzlab.ucr.edu/scripts/wheel/wheel.cgi).

### 3.8. CD Spectroscopy of Synthetic Peptides

The secondary structure of synthetic peptides in membrane mimic solution (1.6 mM SDS, dissolved in 10 mM PBS, pH 7.4) was evaluated as in our previous method [25] using a Chirscan circular dichroism spectrometer (Applied Photophysics Ltd., Surrey, UK) at 25 °C according to the following parameters: scanning wavelength from 190 to 260 nm in 1 mm quartz cuvette, scanning speed of 100 nm/min, step size of 1 nm, and 0.5 nm bandwidth. Spectra were corrected for background contributions by subtraction of appropriate blanks. The delta epsilon in degrees was displayed as a function of the wavelength in nanometers.

### 3.9. Physicochemical Property of Synthetic Peptides

The molecular weight (Da), *pI*, net charge and hydrophobicity of peptides were estimated using the PepDraw Tool (http://www.tulane.edu/~biochem/WW/PepDraw/). The GRAVY and instability index of peptides were predicted by ProtParam Tool (http://web.expasy.org/protparam/). Homology searches of peptides were performed using the APD (http://aps.unmc. edu /AP/ main.html) and BLAST program in NCBI non-redundant peptide database (http://www.ncbi.nlm.nih.gov/blast). The secondary structure of synthetic peptides was predicted by the protein sequence analysis tool of Hierarchial Neural Network in NPS^@^ analysis (https://npsa-prabi.ibcp.fr) with SOPMA method [49].

### 3.10. MIC Determination of Synthetic Peptides

The MIC of synthetic peptides against *E. coli* was determined using a twofold microdilution method described in our previous study [18] with slight modifications. In brief, various concentrations of synthetic peptides (50 μL, stock peptide concentration of 4 mg/mL dissolved in distilled water) were added to a sterile 96-well microplate. Then, 50 μL of *E. coli* suspension at logarithmic phase in broth medium were added to every well. After incubation of 24 h at 37 °C, the MIC is defined as the lowest concentration of peptide at which no visible bacterial growth is observed. Furthermore, the growth of *E. coli* cells was measured by the optical density at 630 nm using a SM-800 micro-plate reader (Shanghai Utrao Medical Instrument Co., Ltd., China) after incubation of different concentrations of synthetic peptides. The same volume of distilled water instead of peptide was used in positive control, and no bacteria with peptide was used in negative control. The percentage inhibition of synthetic peptides was calculated according to the Equation (1) described in Section 3.4. The antibacterial effects of nisin A and ε-poly-L-lysine, two commercial peptides used in food preservatives, were compared in the same conditions. Four replicas were performed in each concentration.

### 3.11. Intracellular and Extracellular H_2_O_2_ Formation in Logarithmic Phase of E. coli Induced by Synthetic Peptides

The capacity of synthetic peptide, which induced intracellular and extracellular H_2_O_2_ production in the logarithmic growth phase of *E. coli* cells after treatment of 3 h at 37 °C, was investigated, referring to the method of our previous studies [19]. Briefly, after incubation of 3 h at 37 °C, the mixture was centrifuged at 4000× *g* for 5 min; the supernatants were collected for detection of extracellular H_2_O_2_ content. The remaining pellets were blended with 300 μL of icy saline and ultrasonically treated in ice bath (5 of pulses, 2 min of each pulse) to destroy the cell membrane completely. After centrifugation at 4000× *g* for 5 min, the supernatants were collected and utilized for the assay of intracellular H_2_O_2_ concentration. An equivalent volume of distilled water instead of the synthetic peptide solution incubated with *E. coli* cells at the same conditions was designated as the bare bacteria group. Before and after ultrasonic treatment, the supernatants of the bare bacteria group were collected. In addition, the H_2_O_2_ self-production of synthetic peptides after addition of saline with a ratio of 1:1 (*v*/*v*) treated under the same conditions was measured as the control. The H_2_O_2_ concentrations of all groups were determined using the H_2_O_2_ assay kit. The efflux of K^+^ from *E. coli* cells (the supernatants without ultrasonic treatment) after incubation of synthetic peptides was determined using the K^+^ assay kit and expressed as mmol/L. All determinations were performed in triplicate for each group.

### 3.12. Statistical Analysis

Data are presented as mean ± standard deviation (*n* = 3). Statistical analysis was performed using the SPSS^®^ software (SPSS Statistical Software 19.0, Inc., Chicago, IL, USA). The independent sample *t*-test was used to evaluate the significant differences between two samples (*p* < 0.05 or *p* < 0.01).

## 4. Conclusions

Several short peptides were identified from the active peptidic fractions derived from HAHp(9.0)-G MRPs. The synthesized peptides HGM-Hp1 (FEDQLR), HGM-Hp2 (ALERTF), and HGM-Hp3 (RHPEYAVSVLLR) had different net charges. However, they possessed positive R residue, and a portion of hydrophobic residues in their sequences. After incubation of 3 h at 37 °C with logarithmic growth phase of *E. coli* cells, the synthetic peptides induced dramatic increases of extracellular and/or intracellular H_2_O_2_ accumulation. Significant increases of K^+^ were also detected in HGM-Hp1 and HGM-Hp2 treatments. Our results suggest that HAHp(9.0)-G MRPs are a good source of antibacterial peptides. These identified peptides could have potential use to treat Gram-negative infections in general. Furthermore, H_2_O_2_ production might be a potential cause of the antibacterial activity for these synthetic peptides.

## Figures and Tables

**Table 1:** Identification of peptides in HE-F2-1 and HE-F2-2 using LC-ESI-Q-TOF-MS/MS.

No.	Peptide Sequence	RT ^a^/min	Tag Length	ALC ^b^/%	m/z	z	Mass	Intensity	Local Confidence/%
HE-F2-1	KGTAVPTAAEATAQR	11.31	15	97	491.2670	3	1470.7790	1.44 × 10^6^	96 91 95 90 95 100 100 100 100 100 100 100 100 100 100
	FEDQLR	15.66	6	99	404.2030	2	806.3922	1.32 × 10^7^	100 100 100 98 100 100
	ALERTF	16.13	6	98	368.7024	2	735.3915	2.70 × 10^6^	100 100 100 99 97 96
	SVVMLR	17.11	6	98	352.7097	2	703.4051	2.97 × 10^5^	94 97 100 100 100 100
	LDVLADK	19.64	7	99	387.2238	2	772.4330	1.58 × 10^6^	99 100 100 100 100 100 100
	LLDRLPRPL	22.97	9	98	364.9008	3	1091.6810	5.02 × 10^6^	100 100 100 99 99 98 99 97 98
	EGDALDELR	25.25	9	98	509.2465	2	1016.4770	1.49 × 10^6^	93 95 100 100 100 100 100 100 100
	EAGAEFDKAAEEVKR	28.22	15	97	550.6095	3	1648.8060	3.47 × 10^6^	98 94 95 90 95 99 100 100 100 100 100 100 99 100 100
	MEVLLLER	35.99	8	99	501.7868	2	1001.5580	4.58 × 10^6^	100 100 100 100 100 100 100 100
HE-F2-2	VATVSLPR	23.43	8	98	421.7583	2	841.5021	5.85 × 10^6^	100 100 99 98 99 98 96 98
	RHPEYAVSVLLR	26.20	12	99	720.4086	2	1438.8044	2.39 × 10^8^	99 100 100 100 100 100 99 99 99 100 100 100

^a^ RT is the retention time in LC system connected to ESI-Q-TOF-MS. ^b^ ALC is the average local confidence scores.

**Table 2:** Physiochemical property of synthetic peptides.

Peptides	Measured/Theoretical Weight/Da	*pI* ^a^	Net Charge	Hydrophobicity/(Kcal/mol)	GRAVY ^b^	Instability Index	Secondary Structure
FEDQLR (HGM-Hp1)	807.07/806.3910	4.00	−1	+14.79	−1.400	72.53	Random coil (100%)
ALERTF (HGM-Hp2)	735.85/735.3903	6.65	0	+11.13	−0.050	28.90	Random coil (100%)
RHPEYAVSVLLR (HGM-Hp3)	1438.42/1438.8021	9.53	+1	+14.45	−0.133	73.08	Random coil (100%)

^a^*pI* represents isoelectric point. ^b^ GRAVY means the grand average of hydropathicity. The PepDraw Tool was used to estimate the molecular weight, *pI*, net charge and hydrophobicity of the peptides, available at http://www.tulane.edu/~biochem/WW/PepDraw/. GRAVY and instability index of peptides were predicted using ProtParam Tool (http://web.expasy.org/protparam/). The secondary structure of peptides HGM-Hp1, HGM-Hp2, and HGM-Hp3 were predicted using the program SOPMA available at Network Protein Sequence (NPS)^@^(https://npsa-prabi.ibcp.fr).

## References

[1] Tong Z., Zhang Y., Ling J., Ma J., Huang L., Zhang L. (2014). An in vitro study on the effects of nisin on the antibacterial activities of 18 antibiotics against *Enterococcus faecalis*. PLoS ONE.

[2] Aoki W., Kuroda K., Ueda M. (2012). Next generation of antimicrobial peptides as molecular targeted medicines. J. Biosci. Bioeng..

[3] Patel S., Akhtar N. (2017). Antimicrobial peptides (AMPs): The quintessential ‘offense and defense’ molecules are more than antimicrobials. Biomed. Pharmacother..

[4] Liu Z., Dong S., Xu J., Zeng M., Song H., Zhao Y. (2008). Production of cysteine-rich antimicrobial peptide by digestion of oyster (*Crassostrea gigas*) with alcalase and bromelin. Food Control.

[5] Sila A., Nedjar-Arroume N., Hedhili K., Chataigne G., Balti R., Nasri M., Dhulster P., Bougatef A. (2014). Antibacterial peptides from barbel muscle protein hydrolysates: Activity against some pathogenic bacteria. LWT-Food Sci. Technol..

[6] Jemil I., Abdelhedi O., Nasri R., Mora L., Jridi M., Aristoy M.C., Toldrá F., Nasri M. (2017). Novel bioactive peptides from enzymatic hydrolysate of Sardinelle (*Sardinella aurita*) muscle proteins hydrolysed by *Bacillus subtilis* A26 proteases. Food Res. Int..

[7] Tang W., Zhang H., Wang L., Qian H., Qi X. (2015). Targeted separation of antibacterial peptide from protein hydrolysate of anchovy cooking wastewater by equilibrium dialysis. Food Chem..

[8] Dashper S.G., Liu S.W., Reynolds E.C. (2007). Antimicrobial peptides and their potential as oral therapeutic agents. Int. J. Pept. Res. Ther..

[9] Hodge J.E. (1953). Dehydrated foods, chemistry of browning reactions in model systems. J. Agric. Food Chem..

[10] Chung Y.C., Kuo C.L., Chen C.C. (2005). Preparation and important functional properties of water-soluble chitosan produced through Maillard reaction. Bioresour. Technol..

[11] Liang C.X., Yuan F., Liu F.G., Wang Y.Y., Gao Y.X. (2014). Structure and antimicrobial mechanism of ε-polylysine–chitosan conjugates through Maillard reaction. Int. J. Biol. Macromol..

[12] Wu S., Hu J., Wei L., Du Y., Shi X., Zhang L. (2014). Antioxidant and antimicrobial activity of Maillard reaction products from xylan with chitosan/chitooligomer/glucosamine hydrochloride/taurine model systems. Food Chem..

[13] Lindenmeier M., Faist V., Hofmann T. (2002). Structural and functional characterization of pronyl-lysine, a novel protein modification in bread crust melanoidins showing in vitro, antioxidative and phase I/II enzyme modulating activity. J. Agric. Food Chem..

[14] Monente C., Bravo J., Vitas A.I., Arbillaga L., Peña M.P.D., Cid C. (2015). Coffee and spent coffee extracts protect against cell mutagens and inhibit growth of food-borne pathogen microorganisms. J. Funct. Foods.

[15] Rufian-Henares J.A., Morales F.J. (2008). Antimicrobial activity of melanoidins against *Escherichia Coli*, is mediated by a membrane-damage mechanism. J. Agric. Food Chem..

[16] Rufian-Henares J.A., Cueva S.P. (2009). Antimicrobial activity of coffee melanoidins-a study of their metal-chelating properties. J. Agric. Food Chem..

[17] Mueller U., Sauer T., Weigel I., Pichner R., Pischetsrieder M. (2011). Identification of H_2_O_2_ as a major antimicrobial component in coffee. Food Funct..

[18] Song R., Wei R.B., Zhang B., Wang D.F. (2012). Optimization of the antibacterial activity of half-fin anchovy (*Setipinna taty*) hydrolysates. Food Bioprocess Technol..

[19] Song R., Shi Q., Yang P., Wei R. (2018). In vitro membrane damage induced by half-fin anchovy hydrolysates/glucose Maillard reaction products and the effects on oxidative status in vivo. Food Funct..

[20] Tong G., Du F., Wu W., Wu R., Liu F., Liang Y. (2013). Enhanced reactive oxygen species (ROS) yields and antibacterial activity of spongy ZnO/ZnFe_2_O_4_ hybrid micro-hexahedra selectively synthesized through a versatile glucose-engineered co-precipitation/annealing process. J. Mater. Chem. B.

[21] Hauser C., Müller U., Sauer T., Augner K., Pischetsrieder M. (2014). Maillard reaction products as antimicrobial components for packaging films. Food Chem..

[22] Lin L., Li B.F. (2006). Radical scavenging properties of protein hydrolysates from Jumbo flying squid (*Dosidicus eschrichitii Steenstrup*) skin gelatin. J. Sci. Food Agric..

[23] Ennaas N., Hammami R., Beaulieu L., Fliss I. (2015). Purification and characterization of four antibacterial peptides from protamex hydrolysate of Atlantic mackerel (*Scomber scombrus*) by-products. Biochem. Biophys. Res. Commun..

[24] Lawyer C., Pai S., Watabe M., Borgia P., Mashimo T., Eagleton L., Watabe K. (1996). Antimicrobial activity of a 13 amino acid tryptophan-rich peptide derived from a putative porcine precursor protein of a novel family of antibacterial peptides. FEBS Lett..

[25] Song R., Shi Q.Q., Yang P.Y., Wei R.B. (2017). Identification of antibacterial peptides from Maillard reaction products of half-fin anchovy hydrolysates/glucose via LC-ESI-QTOF-MS analysis. J. Funct. Foods.

[26] Zhu X., Zhang L., Wang J., Ma Z., Xu W., Li J., Shan A. (2015). Characterization of antimicrobial activity and mechanisms of low amphipathic peptides with different α-helical propensity. Acta Biomater..

[27] Zhang J., Movahedi A., Wang X., Wu X., Yin T., Zhuge Q. (2015). Molecular structure, chemical synthesis, and antibacterial activity of ABP-dHC-cecropin A from drury (*Hyphantria cunea*). Peptides.

[28] Kyte J., Doolittle R.F. (1982). A simple method for displaying the hydropathic character of a protein. J. Mol. Biol..

[29] Falla T.J., Karunaratne D.N., Hancock R.E.W. (1996). Mode of action of the antimicrobial peptide indolicidin. J. Biol. Chem..

[30] Genaro R., Zanetti M. (2000). Structural features and biological activities of the cathelicidin-derived antimicrobial peptide. Biopolymers.

[31] Powers J.P.S., Hancock R.E.W. (2003). The relationship between peptide structure and bacterial activity. Peptides.

[32] Kelly S.M., Price N.C. (2000). The use of circular dichroism in the investigation of protein structure and function. Curr. Protein Pept. Sci..

[33] Kelly S.M., Jess T.J., Price N.C. (2005). How to study proteins by circular dichroism. Biochim. Biophys. Acta (BBA)-Proteins Proteom..

[34] Wall J.S., Williams A., Wooliver C., Martin E.B., Cheng X.L., Heidel R.E., Kennel S.J. (2016). Secondary structure propensity and chirality of the amyloidophilic peptide p5 and its analogues impacts ligand binding—In vitro characterization. Biochem. Biophys. Rep..

[35] Yu H., Huang Y., Huang Q. (2009). Synthesis and characterization of novel antimicrobial emulsifiers from ε-Polylysine. J. Agric. Food Chem..

[36] Zhou C., Li P., Qi X., Sharif A.R.M., Poon Y.F., Cao Y., Chang M.W., Leong S.S., Chan-Park M.B. (2011). A photopolymerized antimicrobial hydrogel coating derived from epsilon-poly-l-lysine. Biomaterials.

[37] Li S., Hao L., Bao W., Zhang P., Su D., Cheng Y., Nie L., Wang G., Hou F., Yang Y. (2016). A novel short anionic antibacterial peptide isolated from the skin of Xenopus laevis with broad antibacterial activity and inhibitory activity against breast cancer cell. Arch. Microbiol..

[38] Hilpert K., Elliott M., Jenssen H., Kindrachuk J., Fjell C.D., Körner J., Winkler D.F., Weaver L.L., Henklein P., Ulrich A.S. (2009). Screening and characterization of surface-tethered cationic peptides for antimicrobial activity. Chem. Biol..

[39] Teixeira V., Feio M.J., Bastos M. (2012). Role of lipids in the interaction of antimicrobial peptides with membranes. Prog. Lipid Res..

[40] Kim I.W., Lee J.H., Subramaniyam S., Yun E.Y., Kim I., Park J., Hwang J.S. (2016). *De novo* transcriptome analysis and detection of antimicrobial peptides of the American Cockroach Periplaneta americana (*Linnaeus*). PLoS ONE.

[41] Bucekova M., Juricova V., Marco G.D., Gismondi A., Leonardi D., Canini A., Majtan J. (2018). Effect of thermal liquefying of crystallised honeys on their antibacterial activities. Food Chem..

[42] Ning C., Wang X., Li L., Zhu Y., Li M., Yu P., Zhou L., Zhou Z., Chen J., Tan G. (2015). Concentration ranges of antibacterial cations for showing the highest antibacterial efficacy but the least cytotoxicity against mammalian cells: Implications for a new antibacterial mechanism. Chem. Res. Toxicol..

[43] Liao W., Lai T., Chen L., Fu J., Sreenivasan S.T., Yu Z., Ren J. (2016). Synthesis and characterization of a walnut peptides-zinc complex and its antiproliferative activity against human beast Carcinoma cells through the induction of apoptosis. J. Agric. Food Chem..

[44] Lee H., Hwang J.S., Lee J., Kim J., Lee D.G. (2015). Scolopendin 2, a cationic antimicrobial peptide from centipede, and its membrane-active mechanism. Biochim. Biophys. Acta (BBA)-Biomembr..

[45] Miao J., Liu G., Ke C., Fan W., Li C., Chen Y., Dixon W., Song M., Cao Y., Xiao H. (2016). Inhibitory effects of a novel antimicrobial peptide from kefir against *Escherichia coli*. Food Control.

[46] Riazi S., Dover S.E., Chikindas M.L. (2012). Mode of action and safety of lactosporin, a novel antimicrobial protein produced by *Bacillus coagulans* ATCC7050. J. Appl. Microbiol..

[47] Bougherra F., Dilmi-Bouras A., Balti R., Przybylski R., Adoui F., Elhameur H., Chevalier M., Flahaut C., Dhulster P., Naima N. (2017). Antibacterial activity of new peptide from bovine casein hydrolyzed by a serine metalloprotease of Lactococcus lactis subsp lactis BR16. J. Funct. Foods.

[48] Ma B., Zhang K.Z., Hendrie C., Liang C.Z., Li M., Doherty-Kirty A., Lajoie G. (2003). PEAKS: Powerful software for peptide de novo sequencing by tandem massspectrometry. Rapid Commun. Mass Spectrom..

[49] Combet C., Blanchet C., Geourjon C., Deléage G. (2000). NPS@: Network protein sequence analysis. Trends Biochem. Sci..

